# (2-*tert*-Butyl-5-hy­droxy­methyl-1,3-dioxan-5-yl)methanol

**DOI:** 10.1107/S160053681202541X

**Published:** 2012-06-13

**Authors:** Berenice Vargas, Amelia Olivas, Gerardo Aguirre, Domingo Madrigal

**Affiliations:** a Centro de Graduados e Investigación del Instituto Tecnológico de Tijuana, Apdo. Postal 1166, 22500, Tijuana, B.C., Mexico; b Centro de Ciencias de la Materia Condensada, Universidad Nacional Autónoma de, México. Km. 107 Carretera Tijuana-Ensenada, Ensenada, BC, CP 22800, Mexico

## Abstract

In the title compound, C_10_H_20_O_4_, the dioxane ring adopts a chair conformation. The *tert*-butyl group occupies an equatorial position, and is staggered with respect to the O atoms of the dioxane ring. In the crystal, mol­ecules are connected by O—H⋯O hydrogen-bonds into zigzag chains of *R* ^4^_4_(8) and *R* ^2^_2_(12) ring motifs that run parallel to the *a* axis.

## Related literature

For background information on the synthesis and properties of 1,3-dioxanes, see: Anderson (1967 ▶); Bailey *et al.* (1978 ▶); Juaristi *et al.* (1987 ▶, 1989 ▶); Vázquez-Hernández *et al.* (2004 ▶). For the crystal structure of a similar compound, see: Zhang *et al.* (2010 ▶).
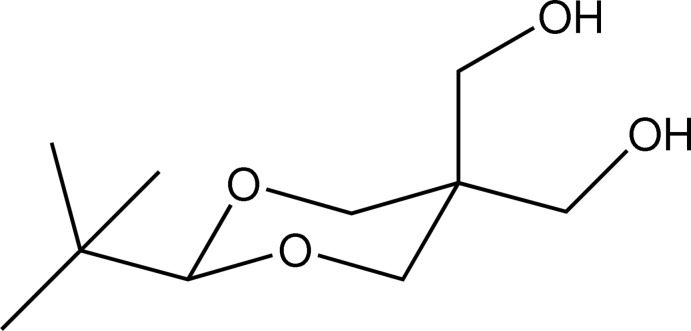


## Experimental

### 

#### Crystal data

C_10_H_20_O_4_
*M* *_r_* = 204.26
Triclinic, 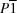

*a* = 5.8337 (10) Å
*b* = 6.1408 (9) Å
*c* = 17.941 (3) Å
α = 81.468 (12)°
β = 87.335 (14)°
γ = 62.606 (13)°
*V* = 564.16 (15) Å^3^
*Z* = 2
Mo *K*α radiation
μ = 0.09 mm^−1^
*T* = 298 K
0.73 × 0.63 × 0.20 mm

#### Data collection

Siemens P4 diffractometer
Absorption correction: empirical (using intensity measurements) (*XEMP* in *SHELXTL*; Sheldrick, 2008 ▶) *T*_min_ = 0.335, *T*_max_ = 0.466
3581 measured reflections
3283 independent reflections
2593 reflections with *I* > 2σ(*I*)
*R*_int_ = 0.013
3 standard reflections every 97 reflections intensity decay: 5.8%

#### Refinement

*R*[*F* ^2^ > 2σ(*F* ^2^)] = 0.054
*wR*(*F* ^2^) = 0.193
*S* = 1.42
3283 reflections
127 parameters
H-atom parameters constrained
Δρ_max_ = 0.35 e Å^−3^
Δρ_min_ = −0.29 e Å^−3^

### 

Data collection: *XSCANS* (Siemens, 1996 ▶); cell refinement: *XSCANS*; data reduction: *XSCANS*; program(s) used to solve structure: *SHELXS97* (Sheldrick, 2008 ▶); program(s) used to refine structure: *SHELXL97* (Sheldrick, 2008 ▶); molecular graphics: *XP* in *SHELXTL* (Sheldrick, 2008 ▶) and *Mercury* (Macrae *et al.*, 2006 ▶); software used to prepare material for publication: *SHELXL97*.

## Supplementary Material

Crystal structure: contains datablock(s) I, global. DOI: 10.1107/S160053681202541X/pk2419sup1.cif

Structure factors: contains datablock(s) I. DOI: 10.1107/S160053681202541X/pk2419Isup2.hkl

Supplementary material file. DOI: 10.1107/S160053681202541X/pk2419Isup3.cml

Additional supplementary materials:  crystallographic information; 3D view; checkCIF report

## Figures and Tables

**Table 1:** Hydrogen-bond geometry (Å, °)

*D*—H⋯*A*	*D*—H	H⋯*A*	*D*⋯*A*	*D*—H⋯*A*
O3—H3*A*⋯O4^i^	0.82	1.94	2.7346 (14)	162
O4—H4*A*⋯O3^ii^	0.82	1.91	2.6878 (15)	159

Symmetry codes: (i) 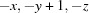; (ii) 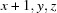.

**Fig. 1.**
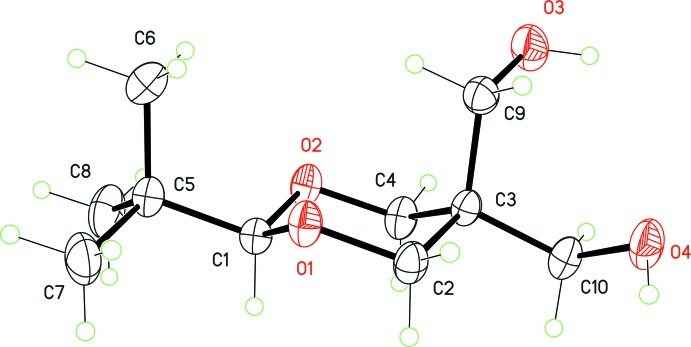
The molecular structure of (I) showing displacement ellipsoids at the 30% probability. H atoms are shown as spheres of arbitrary radius.

**Fig. 2.**
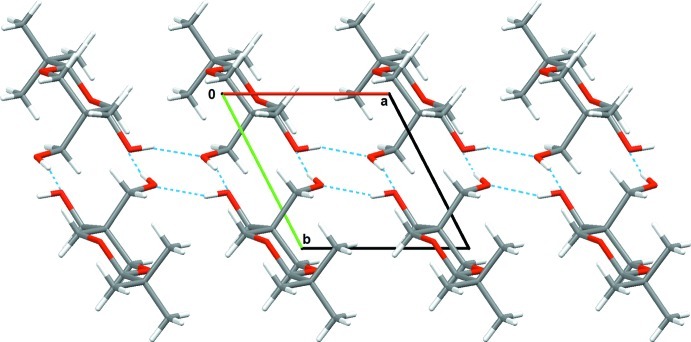
Packing of (I) showing the H-bonds. The molecules are connected into zig-zag ribbons along the [100] direction. H-bonds are indicated by broken lines.

## supplementary crystallographic information

### Comment

The synthesis and conformational studies of several 1,3-dioxanes have been reported in the literature (Vázquez-Hernández *et al.*, 2004; Juaristi *et al.*, 1987, 1989; Bailey *et al.*, 1978). The preparation and some spectroscopic information for the title compound was reported by Anderson *et al.* (1967).

In our development work, while searching rigid molecules to incorporate fluorophore groups, we synthesized 5,5-dihydroxymethyl-2-*tert*-butyl-1,3-dioxane as a reaction intermediate. In the molecule of C_10_H_20_O_4_ (Scheme 1, Fig. 1), the *tert*-butyl group occupies an equatorial position, and is staggered with respect to the O atoms of the dioxane ring. The hydroxyl groups act as both hydrogen bond donor and acceptor to neighboring molecules. The hydrogen-bonds (table 1) form zigzag chains of R^4^_4_(8) and R^2^_2_(12) ring motifs that run parallel to the *a* axis (Fig. 2).

### Experimental

The synthesis of the title compound included reagents and solvents of reagent grade, which were used without further purification. In a 25 ml round bottom flask provided with a magnetic stirrer, was placed 1.2 g (8.8 mmol) of pentaerythritol, 5 ml of water, 0.01 ml of HCl as catalyst and 0.46 ml (7.35 mmol) of pivalaldehyde. 3 ml of dimethylformamide was then added to complete dissolution of the solid, and the reaction mixture was stirred for 24 h. The precipitate thus formed was filtered and washed with a solution of NaHCO_3_ (10ml, 3 times) and H_2_O (10 ml, 3 times). The yield was 41%; melting point: 170–172 °C.

IR(ATR): 3002, 2944, 2969, 2818, 1958, 1479, 1458, 1361, 1166, 1143, 1107, 1038, 1025, 976, 931 cm^-1^.

^1^H-NMR (DMSO-d_6_): δ 4.5 (t, J=5.2 Hz, 1H, O*H*), 4.45 (t, J=5.2 Hz, 1H, O*H*), 3.9 (s, 1H, C*H*), 3.77 (d, J=11.8 Hz, 2H, OCH*H*), 3.5 (d, J=11.8 Hz, 2H, OC*H*H), 3.53 (d, J=5.2 Hz, 2H, C*H*_2_OH), 3.16 (d, J=5.2 Hz, 2H, C*H*_2_OH), 0.8 (s, 9H, C(C*H*_3_)_3_.

^13^C-NMR (DMSO-d_6_): 106.5 (O*C*O), 68.9 (O*C*H_2_), 68.1 (*C*H_2_OH), 59.7 (*C*H_2_OH), 39.0 (*C*), 34.7 (*C*(CH_3_)_3_, 24.6 (C(CH_3_)_3_.

For crystallization, 30 mg of 5,5-dihydroxymethyl-2-*tert*-butyl-1,3-dioxane compound was placed in a glass vial and 2 ml of dimethyl sulfoxide was added. The solution was allowed to stand at room temperature for seven days and the crystals that formed were separated by filtration.

### Refinement

All H atoms were positioned geometrically and refined using a riding model with C—H = 0.96 Å for -CH_3_, C—H = 0.97 Å for -CH_2_- groups and O—H = 0.82 Å. *U*_iso_(H) values were set to 1.2 *U*_eq_(CH_2_) or 1.5 *U*_eq_(CH_3_, OH).

### Crystal data

**Table:**

C_10_H_20_O_4_	*Z* = 2
*M**_r_* = 204.26	*F*(000) = 224
Triclinic, *P*1	*D*_x_ = 1.202 Mg m^−^^3^
Hall symbol: -P 1	Mo *K*α radiation, λ = 0.71073 Å
*a* = 5.8337 (10) Å	Cell parameters from 52 reflections
*b* = 6.1408 (9) Å	θ = 5.6–12.5°
*c* = 17.941 (3) Å	µ = 0.09 mm^−^^1^
α = 81.468 (12)°	*T* = 298 K
β = 87.335 (14)°	Prismatic, colorless
γ = 62.606 (13)°	0.73 × 0.63 × 0.20 mm
*V* = 564.16 (15) Å^3^	

### Data collection

**Table:**

Siemens P4 diffractometer	2593 reflections with *I* > 2σ(*I*)
Radiation source: fine-focus sealed tube	*R*_int_ = 0.013
Graphite monochromator	θ_max_ = 30.0°, θ_min_ = 2.3°
2θ/ω scans	*h* = 0→8
Absorption correction: empirical (using intensity measurements) (*XEMP* in *SHELXTL*; Sheldrick, 2008)	*k* = −7→8
*T*_min_ = 0.335, *T*_max_ = 0.466	*l* = −25→25
3581 measured reflections	3 standard reflections every 97 reflections
3283 independent reflections	intensity decay: 5.8%

### Refinement

**Table:**

Refinement on *F*^2^	Primary atom site location: structure-invariant direct methods
Least-squares matrix: full	Secondary atom site location: difference Fourier map
*R*[*F*^2^ > 2σ(*F*^2^)] = 0.054	Hydrogen site location: inferred from neighbouring sites
*wR*(*F*^2^) = 0.193	H-atom parameters constrained
*S* = 1.42	*w* = 1/[σ^2^(*F*_o_^2^) + (0.1*P*)^2^] where *P* = (*F*_o_^2^ + 2*F*_c_^2^)/3
3283 reflections	(Δ/σ)_max_ < 0.001
127 parameters	Δρ_max_ = 0.35 e Å^−^^3^
0 restraints	Δρ_min_ = −0.29 e Å^−^^3^

### Special details

**Table:**

Geometry. All e.s.d.'s (except the e.s.d. in the dihedral angle between two l.s. planes) are estimated using the full covariance matrix. The cell e.s.d.'s are taken into account individually in the estimation of e.s.d.'s in distances, angles and torsion angles; correlations between e.s.d.'s in cell parameters are only used when they are defined by crystal symmetry. An approximate (isotropic) treatment of cell e.s.d.'s is used for estimating e.s.d.'s involving l.s. planes.
Refinement. Refinement of *F*^2^ against ALL reflections. The weighted *R*-factor *wR* and goodness of fit *S* are based on *F*^2^, conventional *R*-factors *R* are based on *F*, with *F* set to zero for negative *F*^2^. The threshold expression of *F*^2^ > σ(*F*^2^) is used only for calculating *R*-factors(gt) *etc*. and is not relevant to the choice of reflections for refinement. *R*-factors based on *F*^2^ are statistically about twice as large as those based on *F*, and *R*- factors based on ALL data will be even larger.

### Fractional atomic coordinates and isotropic or equivalent isotropic displacement parameters (Å^2^)

**Table:**

	*x*	*y*	*z*	*U*_iso_*/*U*_eq_	
O1	0.19141 (17)	0.04289 (16)	0.28059 (4)	0.0416 (2)	
O2	−0.01630 (17)	−0.14442 (16)	0.22771 (4)	0.0413 (2)	
O3	−0.31024 (19)	0.4084 (2)	0.08767 (6)	0.0652 (4)	
H3A	−0.2724	0.4774	0.0500	0.098*	
O4	0.2736 (2)	0.3620 (2)	0.05181 (5)	0.0572 (3)	
H4A	0.4138	0.3393	0.0675	0.086*	
C1	0.1581 (2)	−0.1720 (2)	0.28510 (6)	0.0374 (3)	
H1A	0.3254	−0.3144	0.2793	0.045*	
C2	0.2949 (2)	0.0938 (2)	0.21038 (6)	0.0422 (3)	
H2A	0.4652	−0.0414	0.2051	0.051*	
H2B	0.3125	0.2437	0.2095	0.051*	
C3	0.1194 (2)	0.12605 (19)	0.14454 (6)	0.0336 (2)	
C4	0.0755 (3)	−0.1038 (2)	0.15426 (6)	0.0439 (3)	
H4B	−0.0495	−0.0829	0.1165	0.053*	
H4C	0.2363	−0.2477	0.1466	0.053*	
C5	0.0515 (2)	−0.2160 (2)	0.36206 (6)	0.0417 (3)	
C6	−0.2078 (3)	0.0058 (3)	0.37302 (8)	0.0546 (3)	
H6A	−0.3292	0.0278	0.3347	0.082*	
H6B	−0.1852	0.1523	0.3694	0.082*	
H6C	−0.2717	−0.0234	0.4218	0.082*	
C7	0.2463 (3)	−0.2507 (3)	0.42305 (8)	0.0663 (4)	
H7A	0.2672	−0.1033	0.4196	0.099*	
H7B	0.4096	−0.3887	0.4158	0.099*	
H7C	0.1839	−0.2816	0.4719	0.099*	
C8	0.0168 (4)	−0.4500 (3)	0.36665 (10)	0.0688 (5)	
H8A	−0.1058	−0.4263	0.3285	0.103*	
H8B	−0.0452	−0.4824	0.4154	0.103*	
H8C	0.1796	−0.5881	0.3589	0.103*	
C10	0.2505 (3)	0.1391 (3)	0.06975 (7)	0.0467 (3)	
H10A	0.4212	−0.0014	0.0720	0.056*	
H10B	0.1517	0.1273	0.0299	0.056*	
C9	−0.1367 (2)	0.3603 (2)	0.14803 (7)	0.0433 (3)	
H9A	−0.2177	0.3427	0.1954	0.052*	
H9B	−0.1020	0.5009	0.1468	0.052*	

### Atomic displacement parameters (Å^2^)

**Table:**

	*U*^11^	*U*^22^	*U*^33^	*U*^12^	*U*^13^	*U*^23^
O1	0.0540 (5)	0.0534 (5)	0.0310 (4)	−0.0366 (4)	−0.0015 (3)	−0.0038 (3)
O2	0.0566 (5)	0.0497 (5)	0.0326 (4)	−0.0369 (4)	0.0046 (3)	−0.0080 (3)
O3	0.0482 (5)	0.0904 (8)	0.0554 (6)	−0.0377 (5)	−0.0154 (4)	0.0201 (5)
O4	0.0621 (6)	0.0766 (7)	0.0491 (5)	−0.0501 (5)	−0.0003 (4)	0.0073 (4)
C1	0.0408 (5)	0.0386 (5)	0.0343 (5)	−0.0200 (4)	0.0022 (4)	−0.0036 (4)
C2	0.0432 (6)	0.0585 (7)	0.0354 (5)	−0.0339 (5)	−0.0020 (4)	0.0003 (5)
C3	0.0378 (5)	0.0412 (5)	0.0294 (5)	−0.0245 (4)	0.0032 (4)	−0.0053 (4)
C4	0.0638 (7)	0.0475 (6)	0.0339 (5)	−0.0357 (6)	0.0066 (5)	−0.0119 (4)
C5	0.0498 (6)	0.0455 (6)	0.0339 (5)	−0.0264 (5)	0.0042 (4)	−0.0027 (4)
C6	0.0543 (7)	0.0636 (8)	0.0489 (7)	−0.0281 (6)	0.0139 (6)	−0.0168 (6)
C7	0.0694 (9)	0.0933 (12)	0.0373 (6)	−0.0425 (9)	−0.0085 (6)	0.0083 (7)
C8	0.1062 (13)	0.0573 (8)	0.0570 (8)	−0.0518 (9)	0.0223 (8)	−0.0066 (6)
C10	0.0540 (7)	0.0600 (7)	0.0355 (5)	−0.0345 (6)	0.0090 (5)	−0.0076 (5)
C9	0.0411 (6)	0.0465 (6)	0.0426 (6)	−0.0218 (5)	0.0004 (5)	−0.0012 (4)

### Geometric parameters (Å, º)

**Table:**

O1—C1	1.4100 (13)	C4—H4C	0.9700
O1—C2	1.4266 (13)	C5—C6	1.529 (2)
O2—C1	1.4181 (14)	C5—C8	1.5307 (19)
O2—C4	1.4301 (13)	C5—C7	1.5356 (19)
O3—C9	1.4204 (15)	C6—H6A	0.9600
O3—H3A	0.8200	C6—H6B	0.9600
O4—C10	1.4248 (16)	C6—H6C	0.9600
O4—H4A	0.8200	C7—H7A	0.9600
C1—C5	1.5267 (15)	C7—H7B	0.9600
C1—H1A	0.9800	C7—H7C	0.9600
C2—C3	1.5297 (15)	C8—H8A	0.9600
C2—H2A	0.9700	C8—H8B	0.9600
C2—H2B	0.9700	C8—H8C	0.9600
C3—C10	1.5235 (15)	C10—H10A	0.9700
C3—C9	1.5314 (16)	C10—H10B	0.9700
C3—C4	1.5309 (15)	C9—H9A	0.9700
C4—H4B	0.9700	C9—H9B	0.9700
			
C1—O1—C2	112.27 (8)	C6—C5—C7	109.52 (12)
C1—O2—C4	111.54 (9)	C8—C5—C7	110.10 (12)
C9—O3—H3A	109.5	C5—C6—H6A	109.5
C10—O4—H4A	109.5	C5—C6—H6B	109.5
O1—C1—O2	110.58 (8)	H6A—C6—H6B	109.5
O1—C1—C5	109.07 (9)	C5—C6—H6C	109.5
O2—C1—C5	109.32 (9)	H6A—C6—H6C	109.5
O1—C1—H1A	109.3	H6B—C6—H6C	109.5
O2—C1—H1A	109.3	C5—C7—H7A	109.5
C5—C1—H1A	109.3	C5—C7—H7B	109.5
O1—C2—C3	110.78 (8)	H7A—C7—H7B	109.5
O1—C2—H2A	109.5	C5—C7—H7C	109.5
C3—C2—H2A	109.5	H7A—C7—H7C	109.5
O1—C2—H2B	109.5	H7B—C7—H7C	109.5
C3—C2—H2B	109.5	C5—C8—H8A	109.5
H2A—C2—H2B	108.1	C5—C8—H8B	109.5
C10—C3—C2	110.54 (9)	H8A—C8—H8B	109.5
C10—C3—C9	111.37 (9)	C5—C8—H8C	109.5
C2—C3—C9	108.81 (9)	H8A—C8—H8C	109.5
C10—C3—C4	108.60 (9)	H8B—C8—H8C	109.5
C2—C3—C4	106.80 (9)	O4—C10—C3	112.63 (10)
C9—C3—C4	110.62 (9)	O4—C10—H10A	109.1
O2—C4—C3	111.25 (9)	C3—C10—H10A	109.1
O2—C4—H4B	109.4	O4—C10—H10B	109.1
C3—C4—H4B	109.4	C3—C10—H10B	109.1
O2—C4—H4C	109.4	H10A—C10—H10B	107.8
C3—C4—H4C	109.4	O3—C9—C3	112.71 (10)
H4B—C4—H4C	108.0	O3—C9—H9A	109.0
C1—C5—C6	110.31 (10)	C3—C9—H9A	109.0
C1—C5—C8	108.76 (10)	O3—C9—H9B	109.0
C6—C5—C8	109.86 (12)	C3—C9—H9B	109.0
C1—C5—C7	108.27 (10)	H9A—C9—H9B	107.8
			
C2—O1—C1—O2	−60.72 (11)	O1—C1—C5—C6	59.66 (13)
C2—O1—C1—C5	179.03 (9)	O2—C1—C5—C6	−61.36 (12)
C4—O2—C1—O1	60.20 (11)	O1—C1—C5—C8	−179.79 (11)
C4—O2—C1—C5	−179.71 (8)	O2—C1—C5—C8	59.20 (14)
C1—O1—C2—C3	58.43 (12)	O1—C1—C5—C7	−60.16 (13)
O1—C2—C3—C10	−170.96 (9)	O2—C1—C5—C7	178.82 (10)
O1—C2—C3—C9	66.45 (12)	C2—C3—C10—O4	−69.64 (13)
O1—C2—C3—C4	−52.99 (12)	C9—C3—C10—O4	51.45 (13)
C1—O2—C4—C3	−58.05 (13)	C4—C3—C10—O4	173.51 (10)
C10—C3—C4—O2	172.44 (9)	C10—C3—C9—O3	56.51 (12)
C2—C3—C4—O2	53.21 (13)	C2—C3—C9—O3	178.60 (9)
C9—C3—C4—O2	−65.05 (12)	C4—C3—C9—O3	−64.38 (12)

### Hydrogen-bond geometry (Å, º)

**Table:**

*D*—H···*A*	*D*—H	H···*A*	*D*···*A*	*D*—H···*A*
O3—H3*A*···O4^i^	0.82	1.94	2.7346 (14)	162
O4—H4*A*···O3^ii^	0.82	1.91	2.6878 (15)	159

Symmetry codes: (i) −*x*, −*y*+1, −*z*; (ii) *x*+1, *y*, *z*.
